# An Assessment of Various Challenges Perceived by Dental Students amidst the COVID-19 Pandemic: A Digital Questionnaire Study

**DOI:** 10.3390/bs12020036

**Published:** 2022-02-04

**Authors:** Hawra Al Hamad, Zahra Al Sunni, Fatimah AlMohsin, Yara AlMaimouni, Abdul Khabeer, Muhammad Ashraf Nazir, Imran Farooq

**Affiliations:** 1 College of Dentistry, Imam Abdulrahman Bin Faisal University, Dammam 31441, Saudi Arabia; 2170000178@iau.edu.sa (H.A.H.); 2170006381@iau.edu.sa (Z.A.S.); 2170000712@iau.edu.sa (F.A.); 2 Department of Restorative Dental Sciences, College of Dentistry, Imam Abdulrahman Bin Faisal University, Dammam 31441, Saudi Arabia; Ykalmaimouni@iau.edu.sa; 3 Department of Preventive Dental Sciences, College of Dentistry, Imam Abdulrahman Bin Faisal University, Dammam 31441, Saudi Arabia; manazir@iau.edu.sa; 4 Faculty of Dentistry, University of Toronto, Toronto, ON M5G 1G6, Canada

**Keywords:** COVID-19, education, dental students, dentistry, psychology

## Abstract

The objective of our study was to evaluate dental students’ perception of the challenges faced during the COVID-19 pandemic related to their clinical work, education, performance, online examinations, psychological health, and teamwork. A validated online questionnaire consisting of closed ended questions was sent to all the undergraduate dental students at our institute. Data were collected and analyzed statistically using a chi-square test to compare responses of male with female and junior with senior students. A total of 317 undergraduate dental students (N = 317) participated in this cross-sectional study. The most common challenges perceived by the participants were related to their exam duration (77.3%), patient availability (66.9%), difficulty in understanding online lectures (58.4%), and a fear of losing grades (57.4%). Participants reported that the COVID-19 pandemic affected their performance in the courses (75.4%), teamwork (72.2%), educational aspects (67.5%), and psychological health (51.1%). A significantly greater proportion of female students reported the adverse effects of COVID-19 on their psychological health than male students (*p* = 0.031). Senior students perceived the negative impact of COVID-19 on teamwork significantly more than the junior students (*p* = 0.004). The majority of students reported challenges during the COVID-19 pandemic. Female students and senior students perceived more challenges than their counterparts. Future studies from other institutes of this region are recommended to establish a clearer picture of COVID-19 related challenges faced by dental students.

## 1. Introduction

The coronaviruses are a group of ribonucleic acid (RNA) viruses that target the respiratory epithelial cells and gastrointestinal tract, facilitating transmission through different routes such as air droplets, fomites, and faeces [1,2,3]. The novel coronavirus disease 2019 (COVID-19) was first identified in Wuhan, China, which then spread to the rest of the world rapidly [3]. It can demonstrate mild symptoms in certain patients (similar to the common cold) while in some, the symptoms can be severe, including shortness of breath, chills, fatigue, nausea, vomiting, and diarrhea [3]. This virus transmits primarily through the droplets of the mouth or nose during breathing, coughing, and sneezing [3,4]. Dental practitioners and dental students who work in close vicinity to the patient’s mouth are at a high risk of infection transmission [5,6]. Therefore, it would be pertinent to assess the dental students’ awareness and attitude towards COVID-19 and develop a strategy to overcome any concerns [7].

Dental education plays an essential role in training and improving infection control measures and knowledge needed to prevent the spread of COVID-19 infection [8,9]. Due to the rapid spread of this disease, urgent strict regulations were established by the World Health Organization (WHO), which affected various life aspects and interfered with numerous routines, including shutting down educational institutions worldwide [3]. According to the social distancing concept, most education providers decided to continue their teaching process using various online learning methods [10,11]. One of the approaches was a synchronous way of teaching and learning that included a live interactive lecture, while another approach was the asynchronous method, which was dependent on the students to learn the provided material and discuss it via emails or discussion boards [11]. However, these methods have been reported to result in increased burnout and decreased student interaction and knowledge retention [11]. Consequently, dental students have faced various learning difficulties during the COVID-19 pandemic [12].

Multiple studies have assessed the impact of the COVID-19 pandemic on dental students. In an earlier study from Italy, dental students appreciated the online learning tools; however, they considered the lack of practical training an essential obstacle in their learning [13]. In another study from Riyadh, Saudi Arabia, dental students reported a high fear of treating patients, and many respondents were willing to handle emergency cases only [14]. There is a lack of data regarding the various challenges faced by dental students due to COVID-19 from Dammam, Saudi Arabia. Therefore, this study was aimed at assessing challenges faced by dental students during the current pandemic. These challenges were divided into four major categories: pre-clinical and clinical challenges, educational challenges, performance challenges, and online examination challenges. In addition, we also assessed the effects of the COVID-19 pandemic on a student’s psychological health, educational aspects, performance in courses, and teamwork.

## 2. Methods

### 2.1. Study Design and Participants

This cross-sectional study included undergraduate dental students from the College of Dentistry, Imam Abdulrahman Bin Faisal University (IAU), Dammam, Saudi Arabia. All male and female students from 2nd to 6th years were invited to participate in the study. Students in the undergraduate program spend one year learning basic science courses in a preparatory year program and get enrolled in dentistry courses from the second year till the sixth year. This is why first year students were not included in this study. The students who were registered in the college (2nd to 6th year), were willing to participate in the study voluntarily, and who provided informed consent were included in the study.

### 2.2. Measurement Instrument

The literature on challenges in dental education was searched, and a questionnaire was drafted by consulting similar previously published studies [8,12,15,16,17]. The questionnaire consisted of closed-ended questions assessing the challenges experienced during the COVID-19 pandemic. Four questions evaluated participants’ responses about the effects of the COVID-19 pandemic on psychological health, education, performance in courses, and teamwork. In addition, there were five items in clinical and pre-clinical challenges, five items in educational challenges, six items in performance challenges, and four items in online examination challenges. A team of researchers critically reviewed the questionnaire to ensure the items in the questionnaire measured the challenges related to dental education. They also evaluated if the questionnaire was appropriate and contained relevant items. This process helped ensure the content and face validity of the questionnaire. Finally, the questionnaire was pilot tested among a sample of 20 students, and their data were not included in the study.

### 2.3. Procedure

The college administration facilitated the distribution of questionnaires, which were sent to students through college emails. The questionnaires were sent to 446 dental students (234 females and 212 males). Data were collected in November/December 2020 and a maximum of three reminders were sent to those students who did not respond during these two months. Additionally, the respondents were free to select the option that they perceived as a COVID-19 related challenge.

### 2.4. Ethics Review

The study was approved by the research ethics committee of the College of Dentistry, IAU (EA 202137). The participants were prompted to informed consent with purpose and details of the study. It was clearly stated that participation was voluntary, and there were no negative consequences associated with refusal to participate or withdrawal from the study. The participants were ensured about the privacy and confidentiality of their data. The ethical guidelines of the Declaration of Helsinki were followed during the execution of the study.

### 2.5. Statistical Analysis

Data analysis was performed using the Statistical Package for Social Sciences (IBM SPSS Statistics for Windows, Version 22.0. Armonk, NY, USA: IBM Corp). The descriptive statistics were reported as frequencies and proportions. A chi-square test was performed to compare of male with female students and junior with senior students. The students enrolled in the second and third-year courses were categorized as junior students, while the students enrolled in fourth, fifth, and sixth-year courses were classified as senior students for analysis purposes. A *p*-value of less than 0.05 was considered statistically significant.

## 3. Results

**Figure 1:**
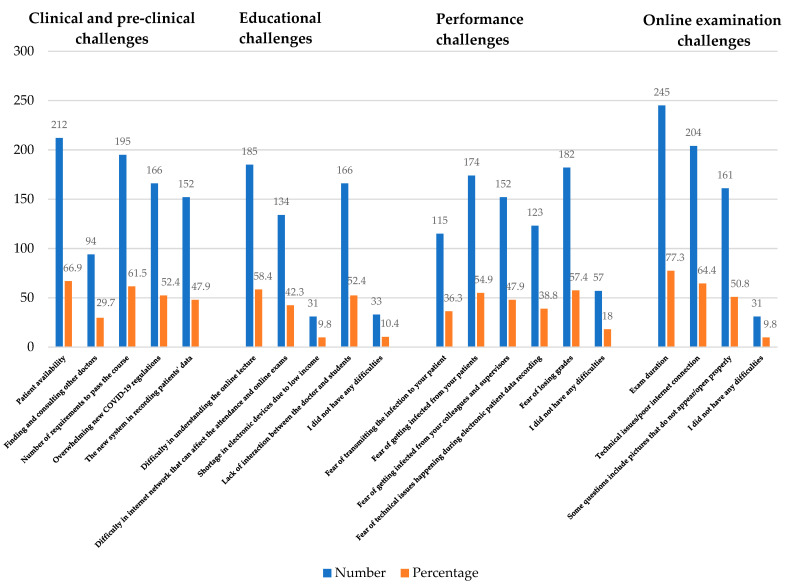
Showing the challenges faced by dental students (in number and percentages) during the COVID-19 pandemic.

In total, 317 students (of the 446 total students) participated in the study, and the response rate was 71%. There was a higher response from females (55.2%) compared to males (44.8%). The majority of the students reported patient availability (66.9%) and the number of requirements to pass the course (61.5%) as the most commonly reported clinical and pre-clinical challenges, whereas consulting the faculty members (29.7%) was the least reported clinical and pre-clinical challenge (Table 1). The most common educational challenges included difficulty in understanding the online lectures (58.4%), and lack of interaction between faculty members and students (52.4%), while only 9.8% reported a shortage in electronic devices due to low income as an educational challenge. Among the performance challenges, the majority of students feared losing grades (57.4%) and feared becoming infected as a result of close interaction with patients (54.9%). The exam duration (77.3%) was the most commonly perceived online examination challenge, followed by technical issues/poor internet connection (64.4%). A detailed representation of the challenges faced by dental students during the COVID-19 pandemic is shown in Figure 1.

A comparison of male and female students’ perceptions about these same challenges is shown in Table 1. There were no significant differences (*p* > 0.05) in male and female students regarding clinical and pre-clinical challenges. Similarly, male and female students did not significantly differ (*p* > 0.05) in their responses about the educational challenges. Among performance challenges, fear of acquiring the infection from their patient was perceived by a significantly greater proportion of females (62.6%) compared to males (37.4%) (*p* = 0.045). Similarly, significantly more female students (61.8%) perceived the risk of getting the infection from colleagues and supervisors (*p* = 0.023) than their male counterparts (38.2%). Statistically significant differences (*p* < 0.05) were found between male and female students regarding online examination challenges. Significantly greater proportions of female students than male students reported difficulties related to exam duration (*p* = 0.004) and technical issues or poor internet connection (*p* = 0.027).

A comparison of junior versus senior students’ perceptions about the same challenges is shown in Table 2. A significantly greater proportion of senior students than junior students reported challenges about patient availability (*p* = <0.001), requirements to pass the course (*p* = <0.001), new COVID-19 regulations (*p* = 0.001), and a new system in recording patients’ data (*p* = <0.001). Educational challenges did not differ significantly (*p* > 0.05) between senior and junior students, although more senior students perceived these challenges. More senior students reported performance challenges related to the fear of transmitting the infection to the patient (*p* = <0.001), fear of becoming infected from patients (*p* = <0.001), fear of becoming infected from colleagues and supervisors (*p* = 0.026), and fear of technical issues during electronic patient data recording (*p* = <0.001). Technical issues/poor internet connection as an online examination challenge was reported by more senior students (72.1%) than the junior students (27.9%), and the difference was statistically significant (*p* = 0.008).

The majority of students reported that COVID-19 affected their performance in the courses (75.4%), teamwork (72.2%), educational aspects (67.5%), and psychological health (51.1%). The comparative analysis of the effects of COVID-19 between male and female students and junior students and senior students was also performed. A significantly greater proportion of female students (61.1%) reported the effects of COVID-19 on psychological health than male students (38.9%) (*p* = 0.031). Significantly more senior students (71.6%) than junior students (28.4%) perceived the effects of COVID-19 on their teamwork (*p* = 0.004).

## 4. Discussion

In our study, the majority of students reported difficulties during the COVID-19 pandemic. The situation related to the COVID-19 pandemic is challenging due to its novelty, high spread rate of the virus, and increased psychological effects [18,19]. As a result, different countries have implemented various measures to control the spread of the disease and its effects. As per recent data, no country has been immune to this virus, and a pandemic of this magnitude has not been seen since the Spanish Influenza [20]. In this regard, dental educational institutes have also been affected severely [6], which has led to multiple challenges faced by dental students, leading to a negative effect on their education, performance, teamwork, and psychological health [21]. Dentistry is taken as a profession by many students, but unfortunately, due to the nature of dental work involving aerosols, dental professionals are more vulnerable to COVID-19 infection [22].

There were 55.2% females and 44.8% males in the present study, which reflects the gender distribution of students enrolled in the dental undergraduate program at our institute. Out of these students, 66.9% reported reduced availability of patients, and 61.5% reported that the number of requirements to pass the course is a major challenge. This finding is in agreement with a study conducted by Kharma et al., which reported that the dental students found working during the pandemic challenging [23]. In the present study, no significant differences were observed between male and female students’ responses regarding clinical and pre-clinical challenges. However, senior students were more affected by these challenges than junior students. Similar findings have been reported recently by Ali et al., who demonstrated in their study that dental students were found to be severely stressed during the COVID-19 pandemic, and amongst this group, senior dental students were found to be more stressed compared with the junior students [24]. This can be attributed to the fact that the senior students work in the clinics and their course requirements are dependent on the availability of patients. In contrast, the junior students work in the laboratory to finish their course requirements, which are non-patient-dependent.

The major challenge reported by dental students related to education was the difficulty in understanding the online lectures (58.4%), followed by the lack of faculty-student interaction during the online lecture (52.4%). Poor voice or video quality and the inability of the lecturer to communicate with the students during the online lecture may have played a role in the origin of this finding, and these are some clear disadvantages of online learning [25]. In addition, dental students also reported the understanding of the online lectures as a major challenge. This finding agrees with a study conducted by Amir et al., which reported that the students had difficulty understanding the online lectures as they found it challenging to maintain focus for a long duration [26].

Regarding the challenges related to students’ performance, overall, 57.4% of the students reported that they have a fear of losing grades. Similar previous studies have also reported that students have expressed their concerns in achieving their learning outcomes and also voiced a fear of losing grades due to the shift to online learning during the COVID-19 pandemic [27,28]. In the present study, 54.9% of students reported fear of becoming infected by their patients, and overall, performance challenges perceived by the females were significantly higher (*p* < 0.05) than males. This can be related to having a higher prevalence of stress, fear, and anxiety in women generally [29]. In our study, the senior students were more significantly affected (*p* < 0.05) compared to junior students in terms of becoming infected with COVID-19, and this could be due to the fact that senior students work in the dental clinics and are aware of the modes of transmission of the virus via droplets. Therefore, they are more concerned about becoming infected.

The duration of online examination (77.3%) was reported as the most common examination challenge, followed by poor internet connection (64.4%). The results of this study are in agreement with the findings of a previous survey conducted by Elsalem in 2020, which reported that the students faced higher stress related to online examinations as they were concerned about the time duration and possibility of encountering technical issues [30]. A recent study from Ireland also reported that one-in-six students at a higher education institution faced broadband coverage problems that could affect their learning [31]. In this regard, the institutions should identify, reach out, and support the students facing this issue. In the present study, statistically significant differences (*p* < 0.05) were found between male and female students regarding online examination challenges. Senior students perceived online examinations more challenging compared to junior students. These findings can be related to the fact that students are not accustomed to online examinations; thus, they fear losing grades [32].

Concerning the limitations of our study, our research represents only dental students at one institute in Saudi Arabia. Perspectives from other institutes could help establish a clear picture of the stressors of the COVID-19 pandemic, as perceived by dental students. Additionally, a limited number of questions were asked to measure the impact of COVID-19 on students, which could also be considered a limitation. Moreover, the questions of this study relied on a self-administration survey; consequently, the interpretations and analyses may have led to a bias compared to face-to-face interviews.

## 5. Conclusions

The majority of students reported the COVID-19 pandemic as a challenge. Female students and senior students perceived more challenges than their counterparts. The students also perceived the effects of the pandemic on their psychological health, education, performance, and teamwork. Dental institutions should address challenges and difficulties arising from the COVID-19 pandemic to provide an effective learning environment for their students.

## Figures and Tables

**Table 1:** Gender differences regarding challenges faced by dental students.

Challenges	Males N (%)	Females N (%)	*p*-Values
**Clinical and pre-clinical challenges**			
Patient availability	97 (45.8)	115 (54.2)	0.625
Finding and consulting other doctors	38 (40.4)	56 (59.6)	0.310
Number of requirements to pass the course	85 (43.6)	110 (56.4)	0.585
Overwhelming new COVID-19 regulations	73 (44.0)	93 (56.0)	0.758
The new system in recording patients’ data	62 (40.8)	90 (59.2)	0.169
**Educational challenges**			
Difficulty in understanding the online lecture	87 (47.0)	98 (53.0)	0.344
Difficulty in internet network that can affect the attendance and online exams	53 (39.6)	81 (60.4)	0.108
Shortage in electronic devices due to low income	17 (54.8)	14 (45.2)	0.236
Lack of interaction between the doctor and students	72 (43.4)	94 (56.6)	0.594
I did not have any difficulties	19 (57.6)	14 (42.4)	0.119
**Performance challenges**			
Fear of transmitting the infection to your patient	43 (37.4)	72 (62.6)	0.045 *
Fear of becoming infected from your patients	71 (40.8)	103 (59.2)	0.115
Fear of becoming infected from your colleagues and supervisors	58 (38.2)	94 (61.8)	0.023 *
Fear of technical issues happening during electronic patient data recording	53 (43.1)	70 (56.9)	0.627
Fear of losing grades	83 (45.6)	99 (54.4)	0.736
I did not have any difficulties	29 (50.9)	28 (49.1)	0.308
**Online examination challenges**			
Exam duration	99 (40.4)	146 (59.6)	0.004 *
Technical issues/poor internet connection	82 (40.2)	122 (59.8)	0.027 *
Some questions include pictures that do not appear/open properly	67 (41.6)	94 (58.4)	0.247
I did not have any difficulties	19 (61.3)	12 (38.7)	0.052

* Statistically significant at *p* < 0.05.

**Table 2:** Differences in challenges faced by junior and senior/clinical dental students.

Challenges	Junior Students N (%)	Senior Students N (%)	*p*-Values
**Clinical and pre-clinical challenges**			
Patient availability	40 (18.9)	172 (81.1)	<0.001 *
Finding and consulting other doctors	25 (26.6)	69 (73.4)	0.109
Number of requirements to pass the course	49 (25.1)	146 (74.9)	<0.001 *
Overwhelming new COVID-19 regulations	41 (24.7)	125 (75.3)	0.001 *
The new system in recording patients’ data	29 (19.1)	123 (80.9)	<0.001 *
**Educational challenges**			
Difficulty in understanding the online lecture	64 (34.6)	121 (65.4)	0.510
Difficulty in internet network that can affect the attendance and online exams	45 (33.6)	89 (66.4)	0.882
Shortage in electronic devices due to low income	13 (41.9)	18 (58.1)	0.272
Lack of interaction between the doctor and students	53 (31.9)	113 (68.1)	0.635
I did not have any difficulties	12 (36.4)	21 (63.6)	0.676
**Performance challenges**			
Fear of transmitting the infection to your patient	19 (16.5)	96 (83.5)	<0.001 *
Fear of becoming infected from your patients	23 (13.2)	151 (86.8)	<0.001 *
Fear of becoming infected from your colleagues and supervisors	41 (27.0)	111 (73.0)	0.026 *
Fear of technical issues happening during electronic patient data recording	22 (17.9)	101 (82.1)	<0.001 *
Fear of losing grades	58 (31.9)	124 (68.1)	0.582
I did not have any difficulties	28 (49.1)	29 (50.9)	0.005 *
**Online examination challenges**			
Exam duration	79 (32.2)	166 (67.8)	0.540
Technical issues/poor internet connection	57 (27.9)	147 (72.1)	0.008 *
Some questions include pictures that do not appear/open properly	52 (32.3)	109 (67.7)	0.751
I did not have any difficulties	14 (45.2)	17 (54.8)	0.134

* Statistically significant at *p* < 0.05.
